# Assessing poisoning risks related to storage of household hazardous materials: using a focus group to improve a survey questionnaire

**DOI:** 10.1186/1476-069X-4-16

**Published:** 2005-08-10

**Authors:** Martin M Kaufman, Susan Smolinske, David Keswick

**Affiliations:** 1 Department of Earth and Resource Science, University of Michigan-Flint, 516 Murchie, Flint, Michigan 48502-1950, USA; 2 Children's Hospital of Michigan, 4160 John R, Detroit, Michigan 48201-2196, USA; 3 Office of Research, University of Michigan Flint, 530 French Hall, Flint, Michigan 48502-1950, USA

## Abstract

**Background:**

In fall of 2004, the authors began an investigation to characterize the correlations between the storage of Household Hazardous Materials and the associated health risks, particularly to children. The study area selected was Genesee County, Michigan, near Flint, with data to be collected by a phone survey of residents and through the acquisition of county hospital records containing procedure codes indicating treatment for poison emergencies, and review of poison control center data.

**Methods:**

A focus group was used to identify key topics and relationships within these data for improving the phone survey questionnaire and its analysis.

**Results:**

The focus group was successful in identifying the key issues with respect to all the data collection objectives, resulting in a significantly shorter and more topically focused survey questionnaire. Execution time of the phone survey decreased from 30 to 12 minutes, and useful relationships between the data were revealed, e.g., the linkage between reading food labels and reading labels on containers containing potentially harmful substances.

**Conclusion:**

Focus groups and their preparatory planning can help reveal data interrelationships before larger surveys are undertaken. Even where time and budget constraints prevent the ability to conduct a series of focus groups, one successful focus group session can improve survey performance and reduce costs.

## Background

In the United States today, the average household stores 3–10 gallons of hazardous materials [1]. Inadvertent exposure to these household hazardous materials (HHMs), along with their improper use and disposal can create health risks. The most recent annual report of the American Association of Poison Control Centers identifies over 2.3 million exposures in 2003. Of these 2.3 million cases, over 50 percent were children under 6 years of age [2]. The Institute of Medicine reported that poisoning is a larger and more important public health hazard than previously realized. They describe 30,800 poisoning-related deaths in 2001, which makes poisoning the second leading cause of injury-related death in the United States. During that same year, there were 282,012 hospitalizations for treatment of poisoning. The estimated annual cost of poisoning was $8.5 billion in 1989, which is equivalent to $12.6 billion in 2003 dollars [3]. For all ages nationwide, the substances most commonly involved in human exposures are analgesics – common painkillers – followed by cleaning substances and cosmetic products. Nationally for children under 6, cosmetics are the most common toxic exposure, followed by cleaning substances and analgesics [2]. Although improvements have occurred in the prevention and treatment of poison emergencies [4-6], the poisoning incidence rate for young children (< 6 yrs.) is still alarming, with the actual rate being close to 4 million annually, since the proportion of incidents reported to Poison Control Centers is estimated to be as low as 26 percent [6].

In fall of 2004, the authors began an investigation to characterize the correlations between the storage of HHMs and the associated health risks, particularly to children. The study area selected was Genesee County, Michigan, near Flint, with data to be collected by a phone survey of residents and through the acquisition of county hospital records containing procedure codes indicating treatment for poison emergencies, and review of poison control center data. Analysis of the survey data would be used to help characterize the storage characteristics of toxic substances within households, and the regional hospital and poison control center call data would be used to help determine if these storage patterns were related to increased visits to hospital emergency rooms. Ultimately, this information would be used to develop intervention strategies for reducing the risks from HHMs to children in Genesee County.

The initial phone survey developed by the research team had 159 questions and in pre-trial testing took 30 minutes to complete. Given project budget constraints, and the strong likelihood respondents would not want to commit 30 minutes of their time to a survey, the research team saw the use of a focus group as an opportunity to shorten – and if possible – improve the survey instrument.

In the public health arena, focus groups have been used to assist research in a wide variety of applications, such as nursing education [7], contraception alternatives [8], and injury prevention among adolescents [9]. Basche [10] defines focus groups as "a qualitative research technique used to obtain data about feelings and opinions of small groups of participants about a given problem, experience, service, or other phenomenon", and they are characterized as an exploratory research method [11]. Exploratory studies typically serve three purposes: (1) to satisfy the researcher's curiosity and desire for better understanding, (2) to test the feasibility of undertaking a more careful study, and (3) to develop the methods to be employed in a more careful study [11].

This paper describes how one focus group held during fall 2004 at the University of Michigan-Flint was used to help refine a phone survey instrument. The topic of the session was "Reducing the Risks to Children from Household Hazardous Materials". Implications for the entire project are also discussed.

## Methods

The methods used in this research were guided by 4 factors: (1) the objectives of the focus group; (2) development of an analytical framework for refining the survey with the focus group results; (3) recruiting the best mix of participants to help achieve the objectives of the focus group; and, (4) developing a favorable environment to ensure the success of the focus group session.

### Focus Group Objectives

Focus groups work particularly well for determining the feelings, attitudes, and manner of thinking of a study population [12]. These characteristics of focus group methodology lend themselves well to the general purpose of improving the content and reducing the length of telephone surveys. The primary purpose of this focus group was to gauge the reaction of the participants to the key terms and concepts used by the researchers and to determine the level of sophistication of participants in differentiating across broad conceptual lines. Specifically, there was concern regarding the ability of members of the study population to differentiate across the concept of "toxicity" as well as the level of "awareness" of the population to the problem. In addition, the terms used in geo-spatial thinking on the part of the population were unknown. By testing the proposed survey questions before a small focus group it was felt that a significant insight could be gained that would allow the researchers to reduce redundancy by focusing on only those terms understood by the population and to rewrite questions in such a way that potential respondents would be able to understand and answer the questions. A brief overview of the development of the phone survey will help provide context for the implementation of the focus group.

The phone survey was designed by the authors – who are an environmental scientist, toxicologist, and research consultant, respectively. A primary objective of this survey was to characterize the storage characteristics of toxic substances within households. Specifically, this meant quantifying the distribution of certain HHMs throughout the different rooms of a home, as well as their storage elevations within each room. The HHMs selected for analysis were the substances involved with the most frequent poison exposures to young children (< 6 years old) as determined by Toxic Exposure Surveillance System (TESS) data for the study area (ibuprofen, acetaminophen, bleach, diaper rash products, acrylic nail products, mouthwash cosmetics, birth control pills, silica gel, vitamins with iron, peroxide, Hg thermometers, prescription medications). For instance, the survey results would yield the relative percentages of ibuprofen stored in the bathroom and bedroom, as well as a general breakdown of its storage elevations (low, below 4 feet; or high, over 4 feet) within each room. It would also be possible to ascertain whether households with young children were storing HHMs at higher elevations. This information would become useful when designing educational materials during the intervention stage of the project.

As noted, the initial phone survey had 159 questions; with the majority involving this 9-question sequence for each of the 13 HHMs listed above: (1) "Do you have this product?"; (2) "How often it was used?"; (3) "Where it was kept?"; (4) "Was the product moved to a new container?"; (5) "Was that container a food container?"; (6) "Has anyone in the house swallowed this substance?"; (7) "How did you respond?"; then, if the respondent did not call the PCC: The interviewer would ask: (8) "Did you consider calling the Poison Control Center?", and, (9) "Was there any particular reason you didn't call the Center?" Although it was not likely questions 2–9 would get asked for every substance, there were potentially 117 (13 * 9) questions possible from this sequence.

The remainder of the survey consisted of: 6 questions which asked respondents to rank certain actions as to whether they constituted a poison emergency (this was the same group of questions used to begin the focus group); 5 questions related to who would be called in the case of a poison emergency (which are redundant with questions 8 and 9 in the sequence listed above); 11 questions which asked how long ago some of the toxic substances listed above had been purchased; 4 questions about the presence and respondents' use of local programs for recycling/collecting hazardous waste; and the 16 remaining questions were divided among ascertaining the respondents' demographic characteristics, whether young children (< 6 yrs.) were present in the household, and the respondents' use and storage of weekly pill planners.

### Development of the Analytical Framework

From this mass of 159 questions, the research team identified 6 general areas of content: (1) assessing the public's general knowledge of what events would constitute a poison emergency; (2) obtaining feedback about the specific products within the home which posed the most risks – especially to children; (3) how people received toxicity information about household hazardous materials; (4) respondents' perception and knowledge of the Poison Control Centers; (5) citizens' involvement with activities such as HHM recycling/pickup programs to reduce the presence of HHMs in their homes; and, (6) storage issues, including where people stored purses, their use and storage of weekly pill planners, and whether containers containing HHMs were changed.

These six content areas became the framework which was used to provide the structure for the questions posed during the focus group, and for the analysis of the participant responses used to change the phone survey. Questions for the focus group were generated within each of the 6 content areas using these guidelines: (1) they would be simple, and without compound construction; (2) the initial questions posed in content area #1 would be designed as "ice breakers" – they would be very straightforward, and act as a transition to the discussion about content group #2; (3) potential linkages between the content groups would be explored by follow-up questions. For example, since education is an important component of each content area, follow-up questions about education would be included throughout the session.

The analysis process using the focus group output was performed systematically. For each of the six content areas, the responses from the focus group were matched to questions of corresponding content in the existing phone survey. Then, the survey questions were evaluated based on these three criteria: (1) were there redundancies present; (2) did the question yield good information; and, (3) did opportunities exist to develop new avenues for analysis.

### Participant Recruitment

While recognizing that focus group methodology is a non-random approach not intended for the purpose of generalization or inference [12], it must also be recognized that a critical requirement for achieving successful exploratory research is "representativeness", which occurs when a sample has the same distribution of characteristics as the population from which it was selected [11]. In this research, the target population consisted of households containing small children. Thus, when recruiting participants for the focus group, most of the effort was directed toward parents of small children and grandparents, since children frequently visit their residences. Since a phone survey was to be conducted throughout the county, there was a need for the ethnic composition of the focus group participants to be representative of the ethnic composition of the broader region. Flyers were placed at the daycare facility on the University of Michigan-Flint's (UM-F) campus, and e-mail notices were sent to university faculty and staff. Since UM-F is a commuter campus, there is a high percentage of non-traditional students (e.g., the average age of the students is over 25). In addition, the university is a major employer in the county, so the constituency of the daycare center provides a good representation of the region's demographics.

After the first attempt during the late summer of 2004 failed to produce enough participants, a second effort mounted in September 2004 using the same recruiting methods yielded 13 participants. A total of 15 participants were initially recruited with the hope of 10 participating. One reasonable explanation for the success of the second effort in September is related to the large numbers of people on vacation in late August. Specific language was used in the publicity campaigns to recruit a mix of parents with young children (under 6 years) and grandparents. The recruitment of grandparents was based on the reasoning that young children often visit their grandparent's homes; by obtaining the characteristics of HHMs within these households it would also provide relevant information for reducing risks to children.

The resulting gender/age/ethnic composition consisted of 11 women and 2 men; with 4 grandparents (3 women, 1 man) and 9 parents of young children (8 women, 1 man). Among the women there were 2 African-Americans, one Hispanic, and 8 whites, while both male participants were white. These characteristics provided a good representation of the ethnicity of the study area, along with a good distribution of young mothers and grandparents.

### Developing a Favorable Environment

Another important factor contributing to a successful focus group is the need to conduct the discussion in a conducive environment [12]. To enable this environment, a central location and convenient time were needed; the location selected was a room at the university with a 4:00 P.M. start time. The university is centrally located in Genesee County, and the start time corresponded to the time many participants picked up their children from the daycare at the university. To help participants through the scheduled two-hour session, pizza and a $50 stipend were also provided.

There were extensive discussions between the project team and staff from the University's Research Office with broad focus group experience regarding the use of video and tape recorders during the session. Unlike focus groups designed to assist the researcher in theory building through the development of rich qualitative data where audio and video recording are essential, focus groups conducted for the purpose of restructuring surveys can be more informal. When the primary purpose of the focus group is to explicate the terms in which participants think note taking and flip charting may be adequate means of recording.

To minimize the potential for bias in the analysis, the decision was made to have two sets of notes transcribed and compared after the session was completed. One person would record notes on a flip chart, and the moderator would also takes notes. The moderator felt that taking notes would help him develop additional questions spontaneously, yet not detract from his effectiveness during the session. University research personnel concurred, noting that while video and audio taping would likely not be intrusive, there were no firm rules for focus group data recording. Another reason for taking this approach was to create pauses at different times during the session to provide breaks. These pauses would allow the moderator to stay on track, and enable participants to confirm exactly what was said – which might not have been discernable from the video or tape recordings after the session was finished.

## Results

A two-hour focus group about reducing the risks to children from household hazardous materials was conducted on September 30, 2004. As participants entered the session, they were requested to voluntarily supply demographic information (parent, grandparent, ethnicity, gender), and all participants provided this information. The session formally opened with the moderator welcoming participants and thanking them for their participation. Notes were taken by personnel from the university's Office of Research and the moderator. No props were used during the session.

Table 1 summarizes the analysis of the phone survey content areas using the evaluation criteria. The high number of "Y" cells in the table indicates that the focus group output provided an effective means for improving the phone survey. A "N/A" entry indicates there were no questions in the phone survey within this content area. Row by row descriptions of how the criteria were used with the focus group responses and applied to the content areas within the phone survey are provided.

**Table 1:** Content area evaluation of the phone survey (N = no, Y = yes, N/A = not applicable)

**Content area**	**Redundancy Present**	**Good Information**	**New Analysis Opportunities**
Poison Emergencies	Y	N	Y
Products posing most risks	Y	N	Y
Acquisition of HHM info	N	N/A	Y
Perception of the PCCs	Y	Y	Y
HHM Recycling	N	Y	Y
Storage Issues	N	Y	Y

### General Knowledge of Poison Emergencies

As an "ice breaker" at the beginning of the session, participants were given a list of 6 questions which asked them to indicate on a scale of 1 to 5 whether exposure to certain substances were "not a poison emergency" (the low end of the scale) or "a very serious poison emergency" (the high end of the scale). The focus group responses indicated good awareness among the participants about the toxicity of swallowing bleach, paint, toadstools, and prescription medications. Swallowing mouthwash and spilling gasoline on someone's shoes were not deemed poison emergencies.

The ability of the participants in 5 out of 6 cases to correctly distinguish between what is and is not a poison emergency (swallowing mouthwash is a poison emergency) indicated there was a good general understanding of the difference between toxic and non-toxic substances. This result led the research team to consider a broader measurement of "awareness" as a better means to understand the public's baseline knowledge in this area. Awareness was conceived as a composite construct consisting of these components: whether a person would call the PCC first in case of a poison emergency; the location within the home for the PCC phone number;, the number of sources used to obtain information about HHMs; and the variety of HHMs (e.g., oil, batteries) taken to a hazardous materials collection center. This last item was included because it indicates a proactive attitude towards a potential hazard.

As indicated across the first row of Table 1, these questions were now redundant (column 1) since other questions fulfilled their role. Nor did they provide good information (column 2), since other questions were deemed more effective for the purpose of assessing existing knowledge of poison emergencies. The responses from the focus group did lead to the development of a new index for measuring awareness (column 3). As a result of this analysis, these six questions were deleted from the phone survey and replaced by four questions related to the composite construct of awareness described above.

### Products Posing the Most Risks

The ice breaking questions were effective in focusing the participants' attention on specific toxic substances, and thus provided an easy transition into a discussion of the products posing the most risk in the home. The unanimous opinion of the participants was that cleaning products were the most toxic and posed the most risk to children because of their perfume-like smell, corrosiveness, and variety. Participants also noted the ingredients list on cleaning products frequently contained warnings about their safety. Vitamins containing iron supplements were mentioned as a threat, but knowledge of these products' toxicity was not well known among the participants.

In response to the follow-up question: "Are there potentially dangerous products which might be confused with non-toxic products – either through their labeling or their taste/smell", the answers were: TUMS^® ^(look like candy); some high blood pressure medication (blue pills look like candy); bubble gum toothpaste; vitamins looking like gummy bears (these do not contain iron); and a newer product called Vitaballs^® ^which looks like bubblegum.

At this juncture, one participant cited the issue of the "perception of parents to toxicity" as being particularly important. A discussion ensued about the levels of toxicity parents were willing to accommodate; e.g., some parents cannot stand cigarette smoke, and thus they are not willing to tolerate this exposure to themselves or their children. Other participants noted some parents were sensitive to food additives, and this sensitivity might translate into poison awareness as these people are more likely to read food labels and other product labels more carefully. Participants agreed there might be a correlation between nutrition consciousness and sensitivity to household hazardous materials (HHMs).

The sequencing of the questions in this content area focused the research team's attention on child safety. There would be increased child safety if access to HHMs was restricted by using locks and storing HHMs at higher elevations in the home. Thus, when the criterion of redundancy was applied, these responses provided the catalyst for consolidating the 9-question sequence used with the 13 toxic substances in the original survey (Table 1, row 2, column 1). The question sequence was: (1) "Do you have this product?"; (2) "How often it was used?"; (3) "Where it was kept?"; (4) "Was the product moved to a new container?"; (5) "Was that container a food container?"; (6) "Has anyone in the house swallowed this substance?"; (7) "How did you respond?"; then, if the respondent did not call the PCC: The interviewer would ask: (8) "Did you consider calling the Poison Control Center?", and, (9) "Was there any particular reason you didn't call the Center?" Although it was not likely questions 2–9 would get asked for every substance, there were potentially 117 (13 * 9) questions possible from this sequence.

As a result of using the framework, the 9-question sequence was reconfigured to contain only 2 questions: "Do you have this substance?" and, "Is this storage location secured by a lock or other device?" To facilitate this consolidation, the revised survey contained this interviewer instruction to respondents: Please indicate where you store the following items (if you don't have an item, please say: "don't have it"). To further cut costs and survey execution time, the substances queried were reduced from 13 to 10 (diaper rash products, acrylic nail products and mouthwash were the three products omitted), thus reducing the questions in the phone survey related to substance storage and access from 117 to 20.

Continuing across the second row, the original questions did not yield good information, since they did not explicitly gather information about storage elevations of HHMs within the home. With better information about storage locations available in the revision, it would now become possible to create an elevation code based upon whether each HHM was stored above or below 4 feet in elevation. Low elevations received a "1", whereas higher elevations received a "2". A composite elevation ratio would also be computable by normalizing the elevations (dividing the total actual elevation score of the items present by the highest potential elevation score). This opened up new avenues for analysis (column 3), as it now became possible to compare this ratio score between households with and without children.

The project team also added this question to the revised survey: "Do you read food labels" to explore the possible correlation between nutrition consciousness and sensitivity to household hazardous materials (HHMs). This item was omitted from the awareness index because the research team wanted to test the responses for correlation independently with the index to help provide confirmation for its validity as an indicator of awareness.

### Acquisition of HHM Information

When participants were asked how they received toxicity information about HHMs, many responded "the Internet", with certain magazines, such as "Mothering" also noted as a source. Some people learned about HHMs and toxic materials from workplace training (food service); others noted they learned from their parents. In the follow-up discussion, most participants said they clearly understood certain substances such as oil, gas, and bleach were toxic. However, participants identified perfume, nail polish, hair products, cosmetics, fabric fresheners, toothpaste, sunscreen, ant/insect sprays, baby oils, hand cleaners with alcohol, and insect repellants with DEET as less threatening substances, with these products often not receiving special precautions in terms of their storage. Participants noted high areas (top shelves) were used for their storage of oil, gas, and bleach.

Participants responded to the next question: "How can we help educate people about the less obvious products which still pose threats within the home?" with: "pediatricians could be more active in their dissemination of information about harmful substances", and by "consistent labels" being placed on these products – a baby's face with a line drawn through it was suggested.

Complacency was seen as an obstacle to parents' education – since many products were used so often – it was difficult to protect children all the time. Participants suggested it might be beneficial to teach children "not to touch anything that is not food". The participants also felt is was more important to educate the parents about HHMs rather than children, since the parents could then teach their children. However, children should also receive HHM education in the schools via videos and guest speakers, as they often do on fire safety.

As shown in Table 1, 3^rd ^row, column 1, there were no redundancies in the original survey since there were no existing questions on this topic. There would also be no way to tell if the information was good. As a result of the discussion in the focus group, we added this open-ended question: "In the past, from what source(s) have you obtained information about potentially harmful materials in the home?". Continuing across the 3^rd ^row, the results would create new analysis opportunities by counting the total sources mentioned, and then including this total as part of the index of awareness.

### Perception of the Poison Control Centers

Discussion was then directed to the perception and knowledge of the Poison Control Centers (PCC). All respondents answered the PCC was not the first place they would call in a poison emergency. After polling the participants, the response ranking was (in order of most to least): pediatrician, emergency room, looking at the label, calling a friend, with the obstacles to calling the PCC being: the phone number was inaccessible; lack of confidence in the "round the clock" and "instantaneous" availability of the PCC services; and possible language barriers. For raising the awareness of PCCs, respondents unanimously supported a suggestion that PCC kits be given out at the time the mother and child are discharged from the birthing center. Midwives could also be given the PCC information packets.

These two questions: "Did you consider calling the Poison Control Center?", and, "Was there any particular reason you didn't call the Center?", were part of the 9-question sequence which was restructured. This accounts for the "Y" in the redundancy criterion (row 3, column 1). The remaining questions pertaining to the PCCs in the existing survey were: In the case of a poison emergency, who would you call first for help?" Who would you call second for help?" Who would you call third for help?", "Where do you keep the number for the Poison Control Center?", and, if the PCC was not mentioned as a place to call, "How would you locate the phone number for the Poison Control Center?".

Responses from the focus group indicated the PCCs were not first in their minds when a poison emergency occurred. This input helped to verify that the existing questions would help assess who people were calling in poison emergencies. Thus, they would yield good information. In terms of new analysis opportunities, two of the questions about PCCs did achieve this as they became part of the awareness index (whether the person would call the PCC first in case of a poison emergency, and the location within the home for the PCC phone number).

### HHM Recycling/Pickup Program Involvement

Participants indicated they received information about HHM recycling from local newsletters (e.g., a local community's newsletter sent to all residents had information about paint recycling). Local TV stations also promoted the biannual hazardous recycling collections in Genesee County. In response to how expired medications were disposed of the participants replied: in the garbage disposal, flushed down the toilet, or thrown in the general garbage pail. No respondents considered taking expired medications back to the pharmacist, and participants were unaware of exchange programs to eliminate mercury thermometers.

The existing survey had four questions related to this content area. As a result of the focus group, these remained unchanged. As shown in row 5 of Table 1, these questions when evaluated were not redundant and yielded good information. The discussion within the focus group about expired medications and mercury thermometers led us to add these questions to the survey: "How do you dispose of expired medications?", and, "If the mercury thermometer broke (assuming the respondent had answered "yes" to having one), how would you dispose of it? No new analysis opportunities resulted from the focus group input in this content area.

### Purse and Weekly Pill Planner Storage, Changing HHM containers

During discussion of these final items, participants stated they stored purses "anywhere" – on tables, on the couch, and hung from the doorknob. When the moderator mentioned the term "weekly pill planners", which was the language used in the existing survey, this term was considered vague by the participants, who suggested "weekly pill dispenser". One respondent (not a senior) noted that childproofing is now available on weekly pill dispensers, but no other respondents (including the seniors) were aware of this capability.

Some participants noted they would change the containers for their pills when they went on vacation, or move liquids from their original spray bottles into old spray bottles (especially cleaning products). Sometimes concentrations of cleaning products were diluted, and this prompted their relocation to another container. Since some medications require refrigeration, one participant asked how these medications could be kept safe from probing children.

Referring to Table 1, 6^th ^row, there were no redundancies in the original survey. The only existing questions on these topics were about weekly pill dispensers: "Does any member of the household use a weekly pill dispenser to dispense their medication?", and, "Do your children ever visit a household where a person uses a weekly pill dispenser (e.g., grandparents)?". We considered these questions adequate and able to yield good information. Based on the discussion in the focus group, these questions related to the transfer of cleaning items were added to the survey: "Have you ever transferred a household cleaning item to another container?", "What item was transferred?", "Was the container labeled?". We also added three similar questions about prescription medications. Finally, we added this question about purse storage: "Where are the purses of the household residents or guests typically placed?"

These new questions offered new analysis opportunities, such as the ability to correlate the relationship between the presence of children and the placement of purses within the home, e.g., were purses stored at higher elevations when children were present? Other avenues of analysis opened up by the focus group discussion were the ability to assess the prevalence of weekly pill dispensers within the study area and perform follow-up during the intervention phase with respect to their locking mechanisms. The discussion also raised the issue of deterring children from accessing refrigerated medications, which is a measure addressable in the intervention phase of the project. And finally, by correcting the terminology used for the pill dispensers, the focus group also helped to improve the survey.

This content area concluded the session. Participants were asked for any additional input, and when none was offered they were thanked for their efforts and again reminded of their capability to contact the project team for additional information about the research.

## Discussion

The focus group was a primary catalyst for improving the phone survey. In effect, the focus group caused the research team to shift the emphasis of the survey away from a detailed substance by substance breakdown to one which considered broader spatial characteristics of HHMs. Specifically, the revised question sequence consisting of room location and elevation allowed investigators to create a spatial coding scheme for the characterization and analysis of toxic substances within the home. It was now possible to obtain a room-by-room accounting of toxic substance storage, and infer the risks to children associated with the substances' relative storage elevations. For example, items stored under the counter, on the floor, or in the drawer were coded as accessible to children; while items stored above the sink, in the medicine cabinet, or any storage location containing "high above" were coded as inaccessible to children.

This coding scheme enables a practical implementation of the "Virtual House" concept used by the U.S. Environmental Protection Agency (EPA) [13]. In the EPA's Virtual House, potentially toxic substances are depicted within each room of the home; this research provides actual substance locations and the associated risks to children. Moreover, the spatial characteristics of toxic substance storage can be compared to regional hospital data to help determine if these storage patterns are related to increased visits to hospital emergency rooms and non-hospitalized poisoning exposures. Ultimately, this information will be useful for developing intervention strategies for reducing the risks from HHMs to children in Genesee County.

### Interdisciplinary Communication

Effective communication between the project team (with training in the natural sciences) and the social scientists from the Office of Research was essential to the success of the project. Underlying this imperative was the reality that the project team had no experience with focus groups (although they had some experience with survey design), while the social scientists knew little about the technical aspects of poison prevention. The communication between the two parties succeeded because of these two actions: (1) before implementation of the focus group, the project team provided the Office of Research personnel copies of the grant proposal, which contained a detailed explanation of the project. This helped acquaint them with the terminology used in poison prevention; (2) over the same period of time, project team members were assigned reading by the Office of Research personnel about focus groups. Meetings were then held to conduct "walkthroughs" of the anticipated focus group session. As noted in this paper, other discussions about taping the session were also held. Overall, communication between the two parties was ongoing and thorough.

### Limitations of Using One Focus Group

There are many concerns about using only one focus group to refine a broader survey instrument. Three concerns come to the forefront: (1) is there adequate representation of the survey's target population?; (2) would there be enough data to help refine the survey?; and, (3) without replication, would the quality of the data suffice? Before conducting the focus group, we did not know the answers to any of these questions. What we concentrated on was recruiting the best mix of participants, and exploiting to good purpose the exploratory nature of the focus group instrument by asking simple open-ended questions in a comfortable environment. These tactics seemed to work.

### Survey Consolidation

The substantial reduction of the questionnaire allowed investigators to insert some new open-ended questions. These questions allowed for the creation of measurement scales, and opened up more capabilities for analysis. The existing questions concerning demographic information, the presence of children, and the respondents' use and storage of weekly pill planners were retained, resulting in a final survey questionnaire containing 54 questions. This final questionnaire took 12 minutes to complete, compared to the 30 minutes needed for the pre-focus group questionnaire.

### Project Implications

As demonstrated by the analysis of the existing survey, we learned that all responses from the focus group were relevant to this survey's refinement. In addition, the focus group responses provided substantial input to other aspects of the project. For example, the suggestion of giving PCC kits out at the time the mother and child are discharged from the birthing center may become one of the strategies attempted during the intervention phase of the project. Giving midwives the PCC information packets may also be attempted.

Analysis of current product labeling is another area where the feedback from the focus group will affect the intervention phase of the project. The responses to the question: "Are there potentially dangerous products which might be confused with non-toxic products – either through their labeling or their taste/smell" may lead to another potential intervention strategy. Here, an attempt may be made to work with local retailers to help identify these substances through the use of a special logo.

## Conclusion

Focus groups are informal, but structured discussions between interested citizens and researchers designed to help the researchers identify key issues within their investigations. Focus groups provide a format of participant interaction that allows for self-disclosure beyond what can be achieved by other methods. This type of meeting format often helps to improve the quality of data collected. In this case, researchers from the University of Michigan-Flint and the Children's Hospital of Michigan solicited input on the characteristics of the harmful materials kept in/around people's homes which might pose risks to young children. The information obtained helped the researchers gain insight into several key issues related to household hazardous materials, consolidate a phone survey and improve the ability to analyze its results. Through follow-up work with citizens and local organizations, this information may also result in the design of more effective intervention strategies for reducing the risks posed to children by harmful substances in the home.

Systematic planning and effective communication must accompany any focus group implementation. Effective planning can help provide not only improved results for specific data collection instruments such as a survey questionnaire, but create general improvement within other project tasks. Effective communication between the project team and the research staff will educate both parties about their roles in the project and help ensure its success.

## List of abbreviations

EPA : Environmental Protection Agency

HHM : Household Hazardous Materials

PCC : Poison Control Center

TESS : Toxic Exposure Surveillance System

UM-F : University of Michigan-Flint

## Competing interests

The author(s) declare that they have no competing interests.

## Authors' contributions

MMK conceived the study, supervised all aspects of its implementation, and led the writing of the manuscript. SS assisted with the study and contributed to the design and analysis of all study components. DK designed the focus group session and assisted with the preparation of the methods section. All authors helped to interpret findings and review drafts of the manuscript.
